# Impact of social determinants of health on perioperative and interstage outcomes in congenital heart surgery a systematic review

**DOI:** 10.3389/fcvm.2026.1881839

**Published:** 2026-09-29

**Authors:** Sara Sakowitz, Jennifer S. Nelson, Justin Robinson, Camilla S. Rossi, David M. Shahian, David T. Cooke, Jeffrey P. Jacobs, Shelby Aughtman, Megan Schultz, Meena Nathan, Claude Beaty, Michal Shafer, Deborah Kozik, Glenn J. Pelletier

**Affiliations:** 1 Department of Surgery, University of California, Los Angeles, CA, United States; 2 Department of Cardiovascular Services, Nemours Children’s Hospital, Orlando, FL, United States; 3 Department of Surgery, University of Central Florida College of Medicine, Orlando, FL, United States; 4 Division of Cardiothoracic Surgery, University of Maryland School of Medicine, Baltimore, MD, United States; 5 Department of Cardiothoracic Surgery, Weill Cornell Medicine, New York, NY, United States; 6 Division of Cardiac Surgery, Department of Surgery, Massachusetts General Hospital, Harvard Medical School, Boston, MA, United States; 7 Division of General Thoracic Surgery, University of California, Davis, Sacramento, CA, United States; 8 Division of Cardiovascular Surgery, Department of Surgery, University of Florida, Gainesville, FL, United States; 9 Department of Surgery, Macon & Joan Brock Virginia Health Sciences at Old Dominion University, Norfolk, VA, United States; 10 Department of Cardiac Surgery, University of Michigan, Ann Arbor, MI, United States; 11 Department of Cardiac Surgery, Boston Children’s Hospital, Boston, MA, United States; 12 Department of Surgery, Harvard Medical School, Boston, MA, United States; 13 Nemours Children’s Health, Delaware Valley, Wilmington, DE, United States; 14 Division of Cardiothoracic Surgery, University of Utah, Salt Lake City, UT, United States; 15 Department of Cardiovascular and Thoracic Surgery, University of Louisville, Louisville, KY, United States; 16 Department of Cardiothoracic Surgery, Stanford School of Medicine, Stanford, CA, United States

**Keywords:** congenital heart disease, congenital heart surgery, interstage outcomes, outcomes disparities, perioperative outcomes, social determinants of health

## Abstract

**Importance:**

Social determinants of health (SDoH)- conditions in which patients live, grow, and age,—are increasingly recognized to influence the health of patients with congenital heart disease (CHD).

**Objective:**

To explore the impact of SDoH on short-term mortality and morbidity, interstage outcomes, and resource utilization, among patients following congenital heart surgery.

**Evidence review:**

A systematic review was completed according to PRISMA guidelines. Relevant studies evaluating the association of SDoH with surgical outcomes of pediatric and adult CHD patients were identified from five databases queried including PubMed, SCOPUS, CINAHL, PYSCINFO, and Cochrane between January 1999 and February 2024. Inclusion criteria incorporated prior systematic reviews and meta-analyses, secondary analyses of prospectively collected trial data, and prospective or retrospective cohort studies. Subsequent reference searches identified 39 related articles. SDoH included patient and neighborhood level metrics. Two independent reviewers screened studies for full-text review, with data extraction by an expert panel.

**Findings:**

Among 1,224 studies identified, 157 articles underwent full-text review and 121 were ultimately selected for data extraction. Eighty-Five articles did not address acute postoperative outcomes and were excluded. Thirty-six studies focused on acute or interstage outcomes following congenital heart surgery and served as the source information. The remainder were retrospective cohort studies using various databases. The number of study participants ranged from 98 to 174,352 (median = 6,979). Factors found to be linked with greater likelihood of in-hospital mortality included Black race, Hispanic/Latino ethnicity, Medicaid insurance, and lower neighborhood socioeconomic status. Both individual and neighborhood level determinants were associated with longer interstage periods, higher complication rates, as well as greater resource utilization and hospital readmissions. Two critical timespans for intervention to reduce inequities in care were the neonatal period and young adulthood.

**Conclusions and relevance:**

SDoH impact both acute and interstage outcomes in patients following surgery for CHD. Developing interventions to increase access to and engagement with quality care and out-of-hospital support may improve both short-term outcomes and reduce systems-level financial burden.

**Systematic Review Registration:**

## Introduction

Congenital heart disease (CHD) represents the most common birth defect, with a prevalence up to 1% of all live births (1). Advances in medical management, surgical technique, and critical care have allowed >97% of patients with CHD to survive into adulthood (2). Despite these improvements, disparities in early morbidity and mortality and long-term survival persist.

A growing body of research across various fields, including cardiac surgery, has explored the influence of social determinants of health (SDoH) on healthcare outcomes and costs. SDoH represent the conditions in which individuals are born, live, work, and age, and may negatively or positively impact health outcomes. They comprise both individual characteristics, such as race, income, or insurance coverage, as well as structural factors, including neighborhood disadvantage or childhood opportunity (3). Prior work has found associations between adverse SDoH and greater disease severity, more frequent emergency presentations, increased healthcare utilization, and poor long-term survival (4–7) (Figure 1). Based on this information, the 2019 American Heart Association and American College of Cardiology guidelines recommend clinicians incorporate social risk in their assessment of cardiac disease (8).

**Figure 1:**
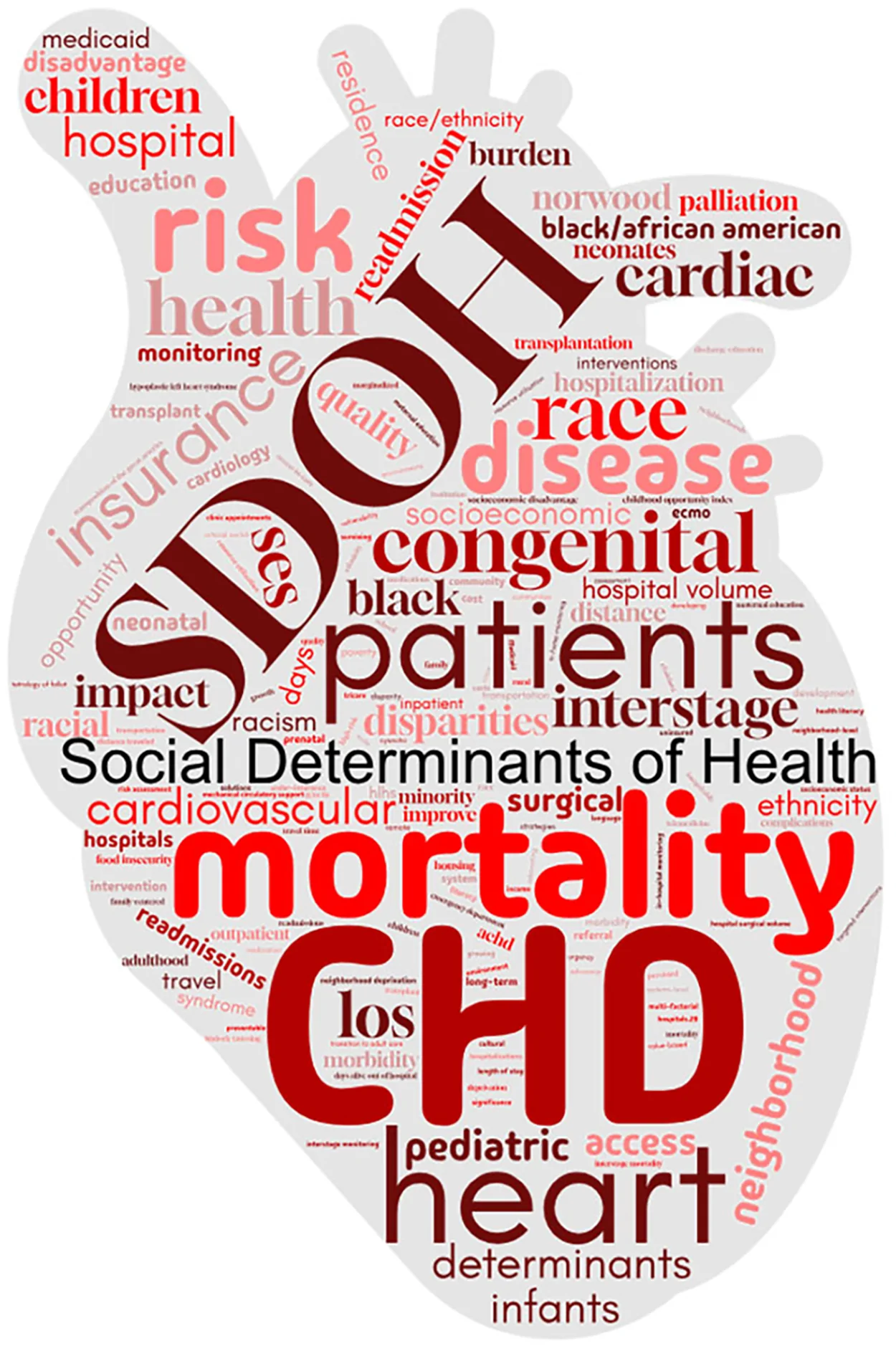
Illustrative depiction of keywords in literature evaluating social determinants of health among congenital heart disease patients. The relative size of each word/phrase reflects the frequency of mention within the reviewed literature and does not indicate the magnitude or strength of any association.

The impact of SDoH on access to care and outcomes may be particularly significant for patients with CHD, given the clinical engagement required for disease management across the lifespan. With the potential to guide efforts to mitigate disparities in health outcomes, a contemporary understanding of the associations between SDoH and outcomes from the neonatal period through adulthood is warranted.

We conducted a comprehensive systematic review to explore the complex and nuanced associations of SDoH with health outcomes in children and adults with CHD. Building on this evidence, we conceptualized interventions to improve outcomes across a lifetime. Our goal was to provide a thorough review of three decades of research on SDoH and offer guidance to health professionals and clinical investigators in addressing disparities at local, regional, and national levels. In this review, we examine the impact of SDoH on perioperative and short-term outcomes.

## Methods

A systematic review was conducted in accordance with the 2020 Preferred Reporting Items for Systematic reviews and Meta-Analyses (PRISMA) guidelines to identify studies that evaluated associations between SDoH and short-term outcomes following congenital heart surgery.

A medical librarian performed a detailed search using search terms in the following databases: PubMed (MEDLINE), Scopus (Elsevier), Cochrane (John Wiley & Sons), CINAHL (EBSCO), and PsycInfo (American Psychological Association). The search was limited to articles published from January 1999 to February 2024. Search results were imported into Covidence for screening. Detailed search strategies are reported in the Supplementary Materials 1. Eligibility for inclusion in this analysis focused on short term and interstage outcomes for patients who underwent congenital heart surgery. Previous reviews were eligible, but none met inclusion criteria for this analysis. A review protocol was not prospectively registered.

The initial search identified 1,518 articles, of which 294 were marked as duplicates. Next, 1,224 studies underwent title and abstract review by two independent reviewers (SS, GP). From this review, 157 articles were identified for full-text review. Eleven authors critically analyzed these publications for content consistent with eligibility criteria, and 121 met inclusion criteria for data extraction. The principal findings in each study were adjudicated by the authors and a decision for inclusion into the systematic review made by consensus. Eighty-Five articles did not address acute postoperative outcomes and were excluded. Thirty-six studies from the US and Canada contained information about associations between SDoH and outcomes in the perioperative or interstage periods following congenital heart surgery and are the focus of this review (Supplementary Figure 1). Study quality was assessed by S.S. and G.P. using the Oxford Center for Evidence-Based Medicine scoring system where 1 indicates the highest level of evidence and 5 the lowest level of evidence (Table 1). Risk of bias was assessed using the Newcastle Ottawa Scale (Supplementary Table 1). The data that support the findings for this study are available from the corresponding author on reasonable request and institutional approval.

**Table 1:** In a comprehensive search, we identified original studies published from 1999 to 2024, that evaluated the impact of social determinants of health (SDoH) on access to and outcomes following congenital heart surgery. Study quality was assessed using the Oxford Center for Evidence-Based Medicine scoring system where 1 indicates the highest level of evidence and 5 the lowest level of evidence.

Reference	Participants	Setting, time period & study design	Key findings	Quality rating
Chan et al. (9)	44,910 CHS recipients ≤18 years undergoing CHS	Retrospective, using KID; 1997–2006	Medicaid insurance and non-White race associated with greater mortality, higher likelihood of emergent admission, and increased odds of referral to high-mortality hospitals.	2
Karamlou et al. (10)	166,599 patients <26 years, including 58,395 CHS recipients	Retrospective, using PHIS; 2016–2020	Median household income and Black race associated with greater mortality. Black neonates experienced longer duration of stay and higher rates of care pathways. Higher and lower income both associated with increased mortality.	2
DiBardino et al. (11)	20,399 CHS recipients <18 years	Retrospective, using the STS-CHSD; 2007–2009	Black race associated with higher mortality, greater morbidity, and longer LOS.	2
Oster et al. (12)	44,017 CHS recipients ≤18 years	Retrospective, using the PHIS; 2004–2008	Non-Hispanic Black race and Hispanic/Latino ethnicity associated with increased mortality, even after adjustment for insurance or operating hospital.	2
Chan et al. (13)	38,801 patients ≤18 years undergoing RACHS procedures	Retrospective, using the KID; 2003, 2006, 2009	Hispanic/Latino ethnicity associated with greater likelihood of experiencing major morbidity, but not FTR. Black race associated with increased odds of FTR and overall mortality.	2
Alabbadi et al. (14)	85,267 CHS recipients <18 years	Retrospective, using the KID; 2009–2019	Black race associated with greater likelihood of FTR. While higher volume hospitals demonstrated lower FTR risk, children of Black race less often underwent surgery at these centers. If Black children underwent surgery at the same hospitals as White patients, race-based disparities would decrease by nearly 50%.	2
Peterson et al. (15)	13,130 patients <18 years undergoing RACHS-1 procedures	Retrospective, using the KID; 2012	Mortality similar across race/ethnicity strata. Government-sponsored insurance associated with greater mortality and longer LOS among neonates. Black race associated with increased LOS among neonates and infants, but not in children >1 year.	2
Bhamidipati et al. (16)	174,352 patients ≥18 years undergoing ACHD operations	Retrospective, using the NIS; 2005–2014	Black patients more frequently underwent urgent/emergent surgery and received less complex operations. Black race associated with similar mortality and complications, relative to White race, upon multivariable adjustment. Among survivors, Black race associated with reduced likelihood of home discharge.	2
DeMone et al. (17)	11,636 CHS recipients <18 years	Retrospective, using state discharge data; 1992, 1996	Medicaid insurance associated with increased mortality odds. Higher mortality institutions had higher proportions of Medicaid insurance cases. Medicaid associated with greater mortality even at low mortality centers.	2
Twedell et al. (18)	549 neonates with SV disease undergoing the Norwood	Secondary Analysis of Prospectively Collected Trial Data, SVR Trial; Enrollment between 2005 and 2008	Lower SES associated with greater mortality hazard up to 4.4 years following the Norwood.	2
Sengupta et al. (19)	478 Norwood recipients	Retrospective single-center; 1997–2017	Patients of lowest SES tertile faced greater risk of early mortality or transplant, longer LOS, greater costs, and higher risk of late mortality or transplant.	2
Tillman et al. (20)	5,748 patients aged 11–64years, who were in active care between 2011 and 2013	Retrospective, using the Colorado CHD surveillance system; 2011–2013	Residence in most deprived areas associated with greater odds of inpatient admission or ED presentation, undergoing cardiac operations, and experiencing major adverse cardiac events. Mortality comparable across ADI strata.	2
Mayourian et al. (21)	6,247 CHS recipients <18 years	Retrospective single-center; 2010–2020	Residence in lower COI areas associated with longer LOS and greater likelihood of major adverse cardiac events or mortality, but similar readmission. Public insurance, caretaker Spanish language were associated with greater mortality.	2
Duong et al. (22)	54,666 patients <18years	Retrospective, using PHIS; 2016–2022	Residence in areas of lowest COI associated with increased likelihood of in-hospital mortality. Non-Hispanic Black race associated with greater mortality on partially adjusted, but similar mortality on fully adjusted models. COI partially mediated the link between race/ethnicity and hospital mortality.	2
Steurer et al. (23)	11,648 infants with cyanotic CHD	Retrospective, using CDC linked birth/death files; 2014–2018	Non-Hispanic Black race and Hispanic/Latino ethnicity associated with significantly greater mortality at 1-year. Adjustment for socioeconomic position accounted for ∼28% of the mortality delta between Non-Hispanic Black and White race and entirely mitigated the disparity for Hispanic/Latino ethnicity.	2
Tjoeng et al. (24)	40,545 CHS recipients <18 years admitted to the intensive care unit	Retrospective, using the Virtual Pediatric Systems database; 2009–2016	African American patients faced higher odds of postoperative mortality. Adjustment for severity of illness mitigated this effect. No consistent race-based trend in illness severity.	2
Williamson et al. (25)	17,275 patients ≤5years with HLHS undergoing the Norwood, Glenn, or Fontan	Retrospective, using the NIS; 2005–2017	Patients at high-volume centers more likely to be of White race, private insurance, and highest income. Increasing hospital volume was associated with incremental increases in the predicted racial disparity index.	2
Chan et al. (26)	130,860 CHS recipients ≤18 years	Retrospective, using PHIS; 2004–2015	Black race associated with increased odds of death and greater risk of mortality without ECMO support. Government insurance associated with increased likelihood of death without ECMO support, and increased mortality following ECMO.	2
Erickson et al. (27)	5,071 CHS recipients <15 years residing in California	Retrospective, using California hospital discharge data; 1992–1994	Managed care insurance associated with lower likelihood of care at low mortality centers, even after adjustment for distance to treating center. Without adjusting for care at lower mortality hospitals, managed care insurance was associated with greater postoperative mortality; this effect was mitigated with adjustment for hospital quality. No association between insurance or care at low mortality centers with LOS.	2
El-Amin et al. (28)	11,748 TRICARE-insured patients ≤17 years	Retrospective, using the US Military Health System Data Repository; 2016–2020	Race not associated with HQH use. Other rank (lowest rank) was associated with significantly lower odds of receiving care at HQH.	2
Morris et al. (29)	463 neonates with HLHS	Retrospective, using Texas Birth Defects Registry Data; 1999–2007	Increasing travel time to a cardiac surgical center associated with significantly greater likelihood of neonatal mortality. Mothers traveling longer distances were of lower education and resided in areas of greater poverty.	2
Olugbuyi et al. (30)	7,711 infants born in Canada, requiring intervention for moderate to severe CHD in the first year	Retrospective, using the Canadian Institute for Health Information- Discharge Abstract Database; 2008–2018	Patients of lowest SES living >300 km had the highest likelihood of mortality in the first year. Patients with severe CHD faced comparable mortality irrespective of distance traveled. Lower SES associated with later age and longer LOS at first intervention.	2
Udine et al. (31)	13,988 infant deaths attributed to CHD	Retrospective, using the US National Center for Health Statistics livebirth and infant death data cohort; 2006–2015	Closer proximity to a top 50 ranked hospital associated with lower mortality risk, as was birth to a mother with a masters, doctorate, or professional degree. Residence in areas of higher poverty associated with greater mortality risk. Each 10% increase in the proportion living in poverty was associated with a 17% increase in mortality risk.	2
Bucholz et al. (32)	525 patients with SV disease undergoing the Norwood	Secondary Analysis of Prospectively Collected Trial Data using the Pediatric Heart Network SVR Trial; 2005–2008	Lowest SES associated with greater 1-year and overall mortality, as well as inferior developmental outcomes. Adjustment for birth characteristics mitigated the association between neighborhood SES and overall mortality, while adjustment for race attenuated the link between SES and neurodevelopment. Patients of low SES had longer interstage periods, faced greater morbidity, and underwent Fontan conversion later, than patients of higher SES. Those of highest SES demonstrated increased likelihood of cardiac transplantation.	2
Ghanayem et al. (33)	426 infants with SV disease who underwent the Norwood and survived to discharge	Secondary Analysis of Prospectively Collected Trial Data using the Pediatric Heart Network SVR Trial; 2005–2008	Hispanic/Latino ethnicity, census block poverty level both independently associated with greater likelihood of interstage mortality.	2
Taylor et al. (34)	273 infants who underwent the Norwood and survived to hospital discharge	Retrospective single-center; 2000–2009	Upon univariate analysis, African American race and residence in census blocks with lower rents or percentages of high school graduates, were associated with greater interstage mortality. These factors were no longer significant on multivariable adjustment.	2
De Loizaga et al. (35)	974 infants undergoing staged SV palliation	Retrospective, using the National Pediatric Cardiology Improvement Collaborative; 2016–2018	The relationship between DI and risk of mortality or transplantation varied, such that residence in areas around median DI was associated with increased hazard, compared to those in DI extremes. Increasing DI was linearly associated with increased LOS and total days hospitalized.	2
Peterson et al. (36)	234 CHS recipients <18 years	Retrospective single-center; 2011–2015	Patients of Black race had an echocardiogram at a later age and were more frequently diagnosed in the ED. After adjustment, race was not associated with increased LOS, greater morbidity, or age at first echocardiogram.	2
Vashist et al. (37)	251 patients ≤18 years undergoing STS benchmark procedures	Retrospective single-center; 2011–2019	Residence in areas of low neighborhood SES associated with longer LOS, only among Norwood recipients. SES only appeared to predict LOS among non-Black patients.	2
Spigel et al. (38)	98 patients with HLHS who underwent the Norwood and survived to discharge	Retrospective single-center; 2008–2018	After adjustment, LOS increased by 4% for each 10-percentile increase in ADI. Insurance, immigration status, and distance traveled were not associated with LOS.	2
Schneider et al. (39)	1,187 CHS recipients ≤18 years	Retrospective single-center; 2015–2018	Increasing DI was associated with greater LOS, but not readmission, among patients in STAT 3, 4, and 5 categories.	2
Sengupta et al. (40)	6,133 CHS recipients <18 years	Retrospective single-center; 2011–2020	Neighborhood COI was not associated with early mortality or transplant. Lower COI was associated with longer LOS, higher costs, and greater adjusted risk of late mortality, transplant, or reintervention.	2
Demianczyk et al. (41)	219 infants undergoing CHS with cardiopulmonary bypass in the first year of life	Retrospective single-center; 2009–2013	Higher social risk associated with a greater number of readmission days and worse adherence to cardiology appointments. Minority race/ethnicity and Medicaid insurance associated with greater readmissions and inferior outpatient appointment adherence.	2
Kim et al. (42)	9,863 patients aged 18–49 years, who underwent adult CHS	Retrospective, using State Inpatient Databases; 2009–2011	Black race, lower median income (<$40,000), and government insurance associated with greater likelihood of readmission.	2
Lushaj et al. (43)	263 CHS recipients	Retrospective single-center; 2011–2015	Higher neighborhood SES and greater distance to the operating center associated with lower likelihood of readmission.	2
Mackie et al. (44)	752 neonates ≤30 days who underwent an arterial switch or Norwood procedure	Retrospective single-center case-control; 1992–2002	Residence in low neighborhood income (<$30,000) areas associated with reduced odds of readmission but may be influenced by greater travel distance.	3

CHS, congenital heart surgery; RACHS, risk adjustment for congenital heart surgery procedures; RVPAS, right ventricle-to-pulmonary artery shunt; MBTS, modified Blalock-Taussing shunt; COI, childhood opportunity index; PIM 2, pediatric index of mortality; PRISM 3, pediatric risk of mortality; PICSIM, pediatric index of cardiac surgical intensive care mortality; ECMO, extracorporeal membrane oxygenation; HLHS, hypoplastic left heart syndrome; LOS, length of stay; STS, society of thoracic surgeons.

Studies were grouped according to the social determinants analyzed in the source literature. Similarities or differences in the findings within groups of SDoH are reported in the results of our review. With the heterogeneity of determinants such as socioeconomic status, the wide range of patient populations and patient ages among studies, and the varied outcomes measured, quantitative pooling was not done.

## Results

### Race & ethnicity

Multiple studies have found an association between Black race or Hispanic/Latino ethnicity and greater mortality risk in both surgical and overall CHD cohorts (9–12). However, at least one work by Peterson et al. (13) found observed race- and ethnicity-based differences in mortality rates in unadjusted analysis, but no statistically significant difference was observed following multivariable adjustment.

Race-based differences in outcomes have been associated with variations in illness severity, such that adjustment for disease complexity mitigates race-based differences in mortality (14). Furthermore, minority race and underinsurance have been associated with greater comorbidity burden, which may contribute to increased procedural and complication risk. Among the ACHD population, for example, Black patients more often presented with greater cardiac disease severity or multi-system comorbidities, yet less frequently underwent complex operations than White patients (15). Other studies indicate both Black race and Medicaid insurance were associated with greater likelihood of emergency hospital admission (9), although it remains unclear whether this finding is related to greater disease severity or reduced access to care.

In addition, Black patients or individuals with public insurance have been documented to less frequently receive care at high surgical volume hospitals (≥233 cases/year) (16). Specifically, patients who underwent palliation for hypoplastic left heart syndrome (HLHS) at high volume centers were found to be more commonly of White race, private insurance, and highest income quartile. Further, as hospital volume increased, the degree of racial disparities similarly grew disproportionately to local demographics (17). As a corollary to this finding, minority race and public insurance have been associated with greater odds of admission to hospitals with high surgical mortality, defined as institutions in the top quintile by either annual standardized mortality rates (9) or hospital-specific mortality rates (18).

Previous studies have suggested unequal access to mechanical circulatory support, such as extracorporeal membrane oxygenation (ECMO), may contribute to postoperative mortality. In one study, however, White and non-White patients had similar rates of ECMO use, but Black patients faced higher mortality odds without ECMO (19). Much of this work has focused on the pediatric population, and the association of race with perioperative morbidity among adult congenital heart disease (ACHD) patients is less clear. However, in one study, Bhamidipati et al. (15) found ACHD patients of Black and White race to demonstrate comparable likelihood of major complications or in-hospital mortality after surgery for CHD.

Additional studies have investigated the association of minority race/ethnicity with higher complication rates and greater likelihood of failure to rescue (FTR), a healthcare quality metric in which failure to recognize and effectively respond to a hospitalized patient experiencing complications results in mortality (16, 20, 21). Despite reports of declining FTR rates among all CHD patients from 2009 to 2019, the likelihood of FTR did not meaningfully change for Black children (20), and Black children and children of other race/ethnicity had higher mortality (i.e., “failure to rescue”) after experiencing a complication.

Evidence from several studies suggests race-based differences in postoperative length of stay (LOS), with Black or Hispanic/Latino patients experiencing significantly longer hospitalizations (11, 12, 20). This association persisted after adjustment for age, sex, insurance type, genetic syndrome, surgical risk category, and type of treating hospital. One investigation suggested this association was present among neonates and infants, but not among children (20). Some single-institution studies have not identified significant associations between race or ethnicity with risk-adjusted LOS (22), potentially due to hospital- or region-specific characteristics or resources. In a study by Vashist et al. (23), race was not independently associated with LOS but acted as an effect modifier for socioeconomic status (SES), which was a significant predictor of hospital LOS.

### Insurance coverage

Relative to private insurance, Medicaid has been associated with greater likelihood of in-hospital mortality (9, 12) and increased FTR (20). This association persisted after risk-adjustment for prematurity, race/ethnicity, surgical complexity, emergency admission status (9), hospital case volume, or overall quality of care, defined by standardized mortality rates (24). However, the available evidence suggests the relationship between insurance and outcomes differs based on age, with one study reporting an association between government insurance and increased mortality odds among neonates, but not patients ≥30 days of age (13).

While some have shown adjustment for hospital mortality rates partially mitigates insurance-based differences in outcomes (25), others have demonstrated that Black race and Medicaid insurance remain associated with greater mortality irrespective of institutional quality (9).

### Socioeconomic disadvantage

In the Pediatric Heart Network Single Ventricle Reconstruction trial, lower composite SES, measured at the census level, was associated with increased risk of mortality in the first year following the Norwood procedure (26). This finding has been reproduced in other studies (27, 28). In one investigation of infants following the Norwood operation, lower SES was associated with greater likelihood of late death or transplantation. Yet, among those who survived the first post-discharge year without requiring transplantation, no association between SES and mortality was identified (27).

Recent work utilized the Childhood Opportunity Index (COI), a composite measure that quantifies neighborhood-level access to resources and conditions that influence child development, including education, health, and socioeconomic factors (29). In one single institution study, residence in low COI areas was associated with greater in-hospital mortality (30). However, this association was not found to be significant at the national level (31).

Patients who have single ventricle (SV) CHD and low SES have been reported to face longer times between the first and second palliative surgeries (interstage period), undergo the Fontan operation later, and experience more complications than those of high SES (32). Meanwhile, in a study of Tricare beneficiaries, lower SES was associated with reduced odds of accessing inpatient care at high quality hospitals (18). Both individual and neighborhood level determinants have been associated with poorer outcomes, and minority race/ethnicity and residence in disadvantaged areas are associated with significantly increased incidence of interstage mortality (33, 34). However, one study found no association between such factors and interstage mortality following multivariable analysis (34), while another investigation detailed an inverse parabolic relationship between neighborhood deprivation and greater hazard of death or transplant (35).

Lower income has also been associated with longer LOS as well as greater hospital cost (13, 27). Several factors may be associated with increased resource utilization, including the need for transfer to a rehabilitation facility, transfer out of state, or preparations for interstage surveillance. However, both public insurance and greater travel burden have not been associated with greater hospitalization duration.

Further, neighborhood disadvantage has been associated with a significant increase in LOS (23, 36), even across Society of Thoracic Surgeons-European Association for Cardio-Thoracic Surgery congenital heart surgery mortality categories (37) or between palliation episodes for SV disease (35). Similarly, residence in low COI areas has been associated with increased postoperative LOS and inpatient costs (38).

Finally, limited work has assessed the influence of the surrounding neighborhood environment on readmission risk. In one study, Mayourian et al. (30) found that areas of low COI were associated with readmission rates within 30 days of discharge that were comparable to those of higher COI areas.

### Travel time or distance

Some investigators have explored access to care through travel time or travel distance. Interestingly, infants with severe CHD tend to live longer distances from treating centers (39). Additionally, mothers of infants who face the largest travel burden, defined as the distance traveled to receive care, are documented to be more commonly of lower education and income (40). However, a study of infants born in Texas with HLHS revealed a positive correlation between longer travel time to a cardiac surgical center and neonatal mortality (41). After risk-adjustment, traveling >90 min to a specialized cardiac center was associated with greater odds of neonatal and pretransfer death. In a Canadian study, patients of low SES faced greater mortality among those residing ≥186.41 miles from the treating hospital (39). Meanwhile, residing in counties near a top 50 ranked pediatric cardiac surgical hospital has been associated with a 11% reduction in risk-adjusted infant mortality. After adjustment for proximity to such institutions, residing in neighborhoods of greater disadvantage remained associated with higher mortality (42).

The relationship between travel distance and readmission risk is complex. Several studies have reported longer travel distance to be associated with lower likelihood of readmission to the index operating center (43, 44). These studies, however, did not capture readmission to other institutions. Mackie and colleagues (43) demonstrated that having a median family income <$30,000 was associated with reduced readmission odds; yet most patients lived >100 miles from the hospital.

### Other factors contributing to differences in outcomes

Underlying SDoH-based differences in outcomes are recognized to be complex. For example, in one study, maternal education and insurance coverage accounted for >28% of race-based differences in 1-year mortality (40).

Among infants undergoing lower mortality risk cardiac operations, increasing social risk, race/ethnicity, primary insurance, maternal age at birth, primary parental language, and distance to cardiac outpatient clinic, were found to be associated with a higher mean number of hospital readmission days (45). Others have identified minority race or ethnicity, government insurance, and unplanned index admission status to be independently associated with greater readmission within 30 days in both pediatric and ACHD cohorts (43, 44, 46).

## Comment

### Principal findings

This systematic review aimed to comprehensively examine the association of individual and neighborhood level SDoH with access to high-quality care and with short term and interstage outcomes for patients following surgery for CHD (Figure 2). Adverse SDoH may be associated with more acute admissions and more severe disease, higher morbidity and mortality rates (including failure to rescue), increased LOS, longer interstage periods, greater resource utilization, and higher rates of hospital readmissions. Single ventricle patients of socioeconomic disadvantage, Black race, or Hispanic/Latino ethnicity experience longer interstage periods and higher interstage mortality.

**Figure 2:**
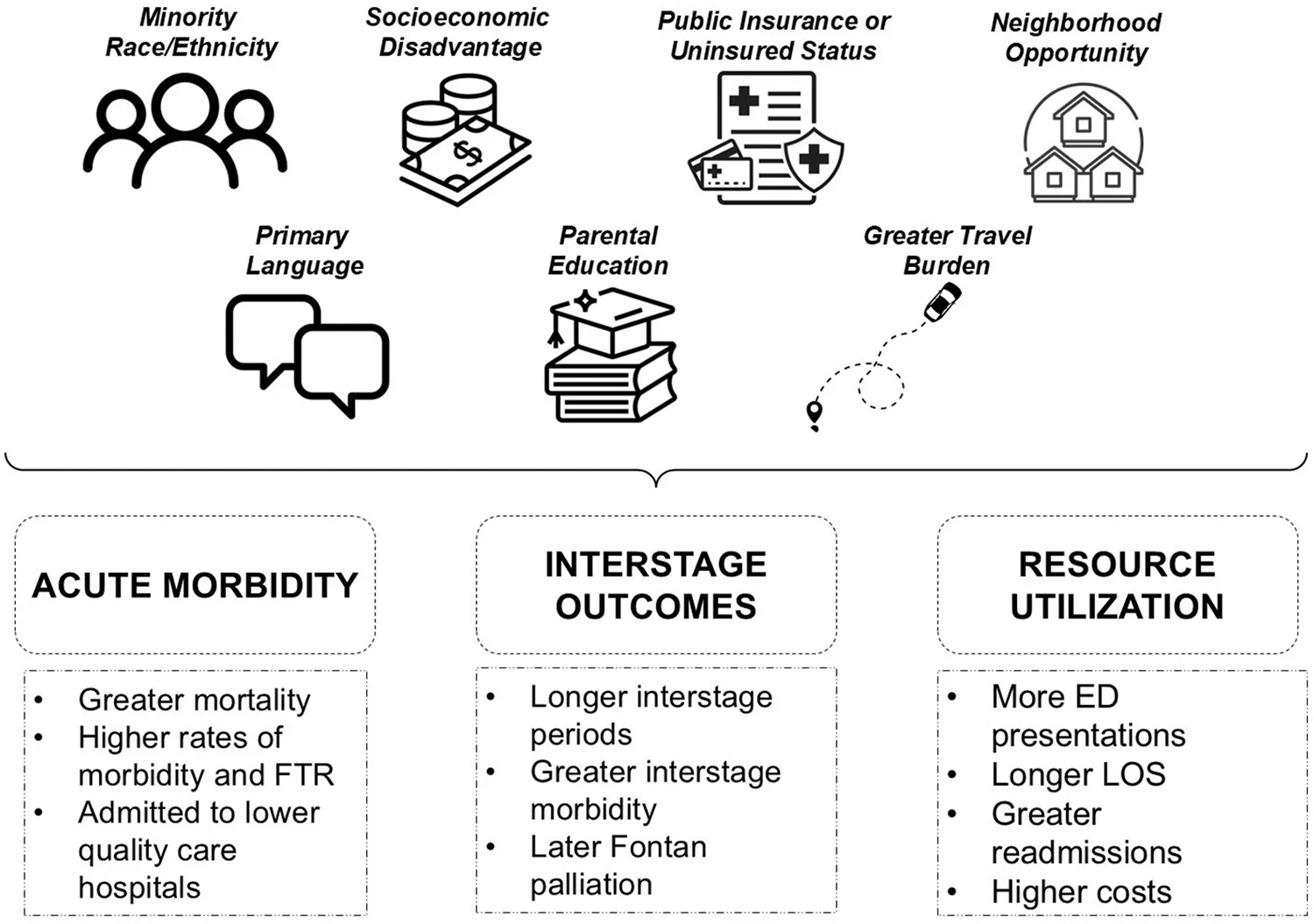
Conceptual summary of reported associations between multi-faceted social determinants of health and acute morbidity/mortality, interstage outcomes, and resource utilization among patients with congenital heart disease (CHD). The figure synthesizes associations described across included studies; it does not imply that every SDoH domain is associated with every outcome or that the reported associations are causal. Different levels of evidence are also noted across individual determinants and outcomes. *FTR, failure to rescue; ED, emergency department; LOS, length of stay.

While race, a social construct based on how individuals identify and ethnicity, an affiliation with a national heritage are often recorded as patient demographics in studies, these characteristics do not confer biologic risk. The associations of race or ethnicity with outcomes in this systematic review likely are based on complex interactions that include structural inequities, socioeconomic factors, healthcare access, disease severity, cultural differences and other cofounders.

Our study demonstrates the association of adverse SDoH with CHD extent and severity at presentation, diminished access to high-quality cardiac surgical centers, worse early and interstage outcomes, and the corresponding need for proactive, directed intervention (Figure 3). These findings support previous statements from the American Heart Association (47) and emphasize the urgency and significance of addressing factors that contribute to these inequitable outcomes.

**Figure 3:**
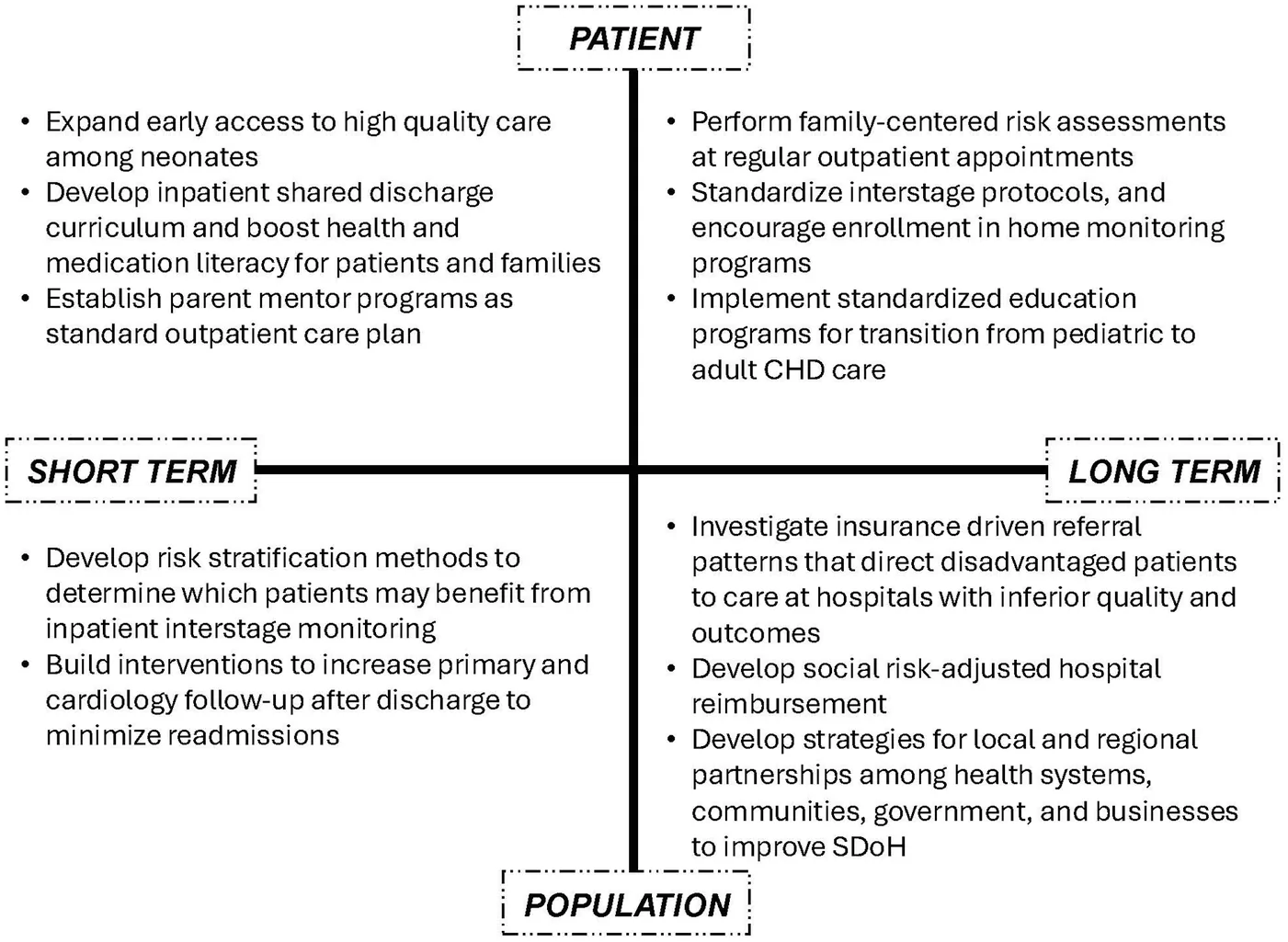
Implementable solutions to address adverse social determinants of health (SDoH) in the short and long-term. The scope of these potential interventions ranges from the individual to the population and are derived from gaps in care identified in the reviewed literature. Rigorous scientific evaluation is needed.

Importantly, the source studies for this systematic review are based on data in the US and Canada. Our findings and the inferences we made from this information guided our suggested interventions to improve short term and interstage outcomes following surgery for CHD in the US. It is likely that these strategies for CHD care may not align precisely with the healthcare systems in other countries, however this framework could be adapted to other situations.

Specific mitigation strategies are discussed below.

### Prenatal and neonatal diagnosis

Neonates are an important group for whom health disparities-focused interventions are needed to expand early access to high quality care. Extensive reports have associated adverse SDoH, especially lack of adequate health insurance, with lower rates of prenatal CHD diagnosis (41, 48, 49). Delayed diagnosis may impede referral for cardiology or operative management, which may lead these patients to face higher symptom burden and higher risk for adverse events upon presentation (9). Such circumstances may increase preoperative risk. While prospective evidence is lacking, expanding prenatal diagnosis particularly in underserved areas, improving CHD screening in newborn nurseries, and prompt referral to high-quality congenital heart surgery centers may help to increase early access to care.

### Lack of access to high quality care

Many have ascribed SDoH differences in outcomes to a lack of access to appropriate care (47). Higher rates of delayed or emergency presentations among minority patients or socioeconomically disadvantaged, potentially due to inadequate access to primary care physicians or cardiologists, may further exacerbate this issue. Even when patients can access care, differences in mortality rates may stem from treatment at high mortality, low quality hospitals (16, 18). In particular, Black children tend to receive congenital heart surgery care in lower volume centers, which also have higher FTR rates (16).

Future studies should investigate referral patterns and insurance networks that specifically aid patients from socioeconomically disadvantaged backgrounds.

### Family and community support mechanisms

Many patients face additive or multi-factorial effects of SDoH (22). Consequently, the most effective interventions may take place long before patients present to the hospital (Figure 3). Improved family health literacy, consistent access to medications, food security, transportation, and stable housing can all shape patient health and symptom severity. Community providers should seek to perform family-centered risk assessments at regular outpatient appointments and connect these families with relevant resources (50). Ongoing evaluations are needed to identify specific factors contributing to increased morbidity associated with SDoH, thereby informing quality improvement programs at various levels.

### Interstage support and monitoring

The interstage period in SV palliation is an exquisitely vulnerable time and presents an excellent opportunity to address SDoH that adversely shape outcomes. In the absence of standardized protocols across centers, considerable variation exists in discharge education, nutrition routes, and home surveillance plans, all of which may underlie differences in interstage events (51). Because many patients are discharged home between staged palliation episodes, interventions have trialed remote monitoring programs (27) and expanded telemedicine utilization (52), with encouraging results.

Shustak et al. (53) developed the SV Management and Monitoring Program, which involved home monitoring, expanded pre-discharge education, nursing visits, regular clinical check-ins, and preemptive scheduling of stage 2 palliation. Enrollment in this initiative completely mitigated the impact of high social vulnerability on interstage weight gain and growth failure. Cherestal and colleagues found no difference in death or transplant listing based on neighborhood SES among infants enrolled in a remote digital home monitoring program (54). These findings suggest broad engagement in monitoring programs may ameliorate SDoH differences in interstage outcomes.

Such efforts are especially important in the modern era of care centralization, where patients of socioeconomic disadvantage may face transportation or distance barriers to interstage care (55). However, home monitoring programs are complex endeavors that rely on active parental engagement, and access to dedicated cardiac teams and hospital or outpatient evaluation. Even among those enrolled, readmission rates have been reported up to 66% (56). Therefore, some have suggested in-hospital monitoring programs might confer benefit, particularly for patients deemed at high-risk for inter-stage mortality (57). Expanded utilization of risk-stratification models may identify patients who could benefit from in-hospital stays between palliation episodes (58).

### Discharge preparation, home support, and preventable readmissions

Finally, in the era of value-based care, efforts to reduce expenditures and decrease readmissions are increasingly important. Hospitalizations for CHD patients comprise over 15% of total pediatric hospitalization costs (59) and have been associated with the highest readmission rate among all pediatric presentations (60). Prior literature has identified factors which may prove appropriate targets to reduce readmissions (61).

Greater disease severity or complexity has been associated with higher readmission risk. While extent of cardiovascular disease is not inherently modifiable, these patients may significantly benefit from greater engagement with outpatient longitudinal care. Efforts could be targeted at increasing access to post-discharge primary and cardiology follow-up, with the goal of maximizing days spent out of hospital.

Some have proposed that improved teaching before discharge may contribute to reductions in hospital re-presentation. Kolwaite and colleagues (29) describe a “Ticket to Home” program, in which parents undergo trainings, obtain all necessary medications and equipment, and demonstrate their ability to independently care for their child before being discharged. The authors found no difference in readmission rates across COI levels, although patients of low COI did face longer postoperative LOS. However, Kim et al. (62) found that, irrespective of readiness at discharge, families of lower SES experienced higher rates of emergency department (ED) presentation and hospital readmission. This is consistent with prior work linking Black race (63) and underinsurance (64) with greater likelihood of ED visits and subsequent hospital admission.

With an increasing number of annual ED visits, novel interventions are needed to better guide patients' outpatient course. Future investigations could evaluate the role of discharge education amongst broader patient populations, including the development of a shared discharge curriculum to educate patients and their families while they are in the hospital. Efforts to improve health and medication literacy may benefit patients' self-management following discharge (65). Furthermore, adolescent patients who face a transition from pediatric to ACHD care remain at high-risk for ED presentation (66) and readmissions (67). In parallel, programs should seek to implement standardized transition education programs, while launching investigations to better understand the precise factors contributing to loss to follow-up (67).

Parent mentors are specially trained healthcare workers with personal experience caring for their own children with specific medical conditions (68). One randomized controlled trial demonstrated improvements in health and reduction in parental workdays missed for children with asthma (68), while another study showed improved access to care and increased insurance coverage for uninsured minority children (69). These results plus cost savings identified with these care plans have led to federal governmental funding to support these programs (70–73). While this evidence is from other US specialty healthcare settings, adoption of a parent mentor system for patients with CHD has the potential to improve outcomes across the spectrum of SDoH.

With governmental programs seeking to reduce readmissions through penalty programs, policy makers should carefully consider the place of social risk-adjusted reimbursement. Without proper adjustment for social factors, hospitals that care for larger proportions of patients with adverse SDoH may face financial penalties (74). In contrast, appropriately risk-adjusted reimbursement could encourage centers to trial innovative care pathways or interventions to improve care outcomes in both the acute and longer-term setting.

### Limitations

There are several limitations to the present work. The evidence is retrospective, does not account for residual confounding, is restricted to studies in the US and Canada, and the ability to infer causality is limited. Several publications used recurring administrative databases or the same prospective cohorts. Therefore, the 36 source articles may not represent 36 separate study populations. Our discussion of SDoH was restricted by the variability in how SDoH constructs were represented in the original literature. Specifically, studies that used neighborhood- or census-level socioeconomic measures as proxies for individual socioeconomic conditions raise the possibility to amplify errors in inferring individual risk. When possible, we attempted to clarify the definition or metric used in each study. Furthermore, when evaluating multivariable model outputs, we could not confirm the degree or efficacy of risk-adjustment. We recognize the potential for collinearity or intersectionality between SDoH, which may have influenced risk-adjustment.

Despite these limitations, this systematic review followed PRISMA guidelines and best practices in literature search and data extraction, and it presents the most comprehensive perspective to date on the association of SDoH with perioperative morbidity and mortality, interstage outcomes, and resource utilization among CHD patients.

## Conclusion

Our study underscores the substantial impact of SDoH (e.g., individual or neighborhood disadvantage, underinsurance, or minority race or ethnicity) on timely diagnosis, disease severity at presentation, access to high-quality care, acute and interstage outcomes, LOS, readmissions, and resource utilization for CHD patients. Critical areas for intervention include the neonatal and interstage periods and the transition to adult cardiac care.

The development of multidisciplinary initiatives to better identify and address factors contributing to delayed diagnosis, lower quality care, and worse acute and interstage outcomes, including the expansion of home monitoring and follow-up programs, have the potential to mitigate these persistent health disparities. Parent mentors may provide a bridge from the complex healthcare system to the social environment for the individual patient with CHD and facilitate optimal care. These endeavors could have transformative positive impacts on the health of these complex patients, while reducing the financial burden on the health system.

## Data Availability

The original contributions presented in the study are included in the article/Supplementary Material, further inquiries can be directed to the corresponding author/s.

## Supplementary material

Supplementary Figure 1 PRISMA study flow diagram starting with 1518 articles published from five databases searched, eliminating irrelevant articles, and concluding with 36 articles that report acute postoperative outcomes following congenital heart surgery.

## References

[1] Hoffman JIE Kaplan S. The incidence of congenital heart disease. J Am Coll Cardiol. (2002) 39(12):1890–900. 10.1016/S0735-1097(02)01886-7 12084585

[2] Mandalenakis Z Giang KW Eriksson P Liden H Synnergren M Wåhlander H. Survival in children with congenital heart disease. J Am Heart Assoc. (2020) 9(22): e017704. 10.1161/JAHA.120.017704 33153356 PMC7763707

[3] World Health Organization. Social determinants of health. Available online at: https://www.who.int/health-topics/social-determinants-of-health (accessed December 2, 2023).

[4] Lopez KN Jordan CAL Moreno J. Sociodemographic indices in pediatric heart failure. J Heart Lung Transplant. (2023) 42(2):156–9. 10.1016/j.healun.2022.10.027 36428204

[5] Churchwell K Elkind MSV Benjamin RM Carson AP Chang EK Lawrence W. Call to action: structural racism as a fundamental driver of health disparities. Circulation. (2020) 142(24):e454–68. 10.1161/CIR.0000000000000936 33170755

[6] Son H Zhang D Shen Y Jaysing A Zhang J Chen Z. Social determinants of cardiovascular health: a longitudinal analysis of cardiovascular disease mortality in US counties from 2009 to 2018. J Am Heart Assoc. (2023) 12(2):e026940. 10.1161/JAHA.122.026940 36625296 PMC9939060

[7] Acquah I Hagan K Javed Z Taha MB Valero-Elizondo J Nwana N. Social determinants of cardiovascular risk, subclinical cardiovascular disease, and cardiovascular events. J Am Heart Assoc. (2023) 12(6):e025581. 10.1161/JAHA.122.025581 36926956 PMC10111542

[8] Hammond G Johnston K Huang K Joynt Maddox KE. Social determinants of health improve predictive accuracy of clinical risk models for cardiovascular hospitalization, annual cost, and death. Circ Cardiovasc Qual Outcomes. (2020) 13(6):e006752. 10.1161/CIRCOUTCOMES.120.006752 32412300 PMC7299736

[9] Chan T Pinto NM Bratton SL. Racial and insurance disparities in hospital mortality for children undergoing congenital heart surgery. Pediatr Cardiol. (2012) 33(7):1026–39. 10.1007/s00246-012-0221-z 22349675

[10] Karamlou T Hawke JL Zafar F Kafle M Tweddell JS Najm HK. Characterizing socioeconomic and racial disparities in congenital heart disease. Ann Thorac Surg. (2022) 113(1):157–65. 10.1016/j.athoracsur.2021.04.008 33872577

[11] DiBardino DJ Pasquali SK Hirsch JC Benjamin DK Kleeman KC Salazar JD. Effect of sex and race on outcome in patients undergoing congenital heart surgery. Ann Thorac Surg. (2012) 94(6):2054–60. 10.1016/j.athoracsur.2012.05.124 22884593 PMC3838919

[12] Oster ME Strickland MJ Mahle WT. Racial and ethnic disparities in post-operative mortality following congenital heart surgery. J Pediatr. (2011) 159(2):222–6. 10.1016/j.jpeds.2011.01.060 21414631

[13] Peterson JK Chen Y Nguyen DV Setty SP. Current trends in racial, ethnic, and healthcare disparities associated with pediatric cardiac surgery outcomes. Congenit Heart Dis. (2017) 12(4):520–32. 10.1111/chd.12475 28544396 PMC5548594

[14] Tjoeng YL Jenkins K Deen JF Chan T. Association between race/ethnicity, illness severity, and mortality in children undergoing cardiac surgery. J Thorac Cardiovasc Surg. (2020) 160(6):1570–1579.e1. 10.1016/j.jtcvs.2020.06.015 32739167

[15] Bhamidipati CM Garcia IC Kim B McGrath LB Khan AM Broberg CS. Racial disparity: the adult congenital heart disease surgery perspective. Pediatr Cardiol. (2024) 45(6):1275–83. 10.1007/s00246-022-03087-5 36580104

[16] Alabbadi S Rowe G Gill G Chikwe J Egorova N. Racial disparities in failure to rescue after pediatric heart surgeries in the US. J Pediatr. (2024) 264:113734. 10.1016/j.jpeds.2023.113734 37739060

[17] Williamson CG Tran Z Rudasill S Hadaya J Verma A Bridges AW. Race-based disparities in access to surgical palliation for hypoplastic left heart syndrome. Surgery. (2022) 172(2):500–5. 10.1016/j.surg.2022.03.017 35450745

[18] El-Amin A Koehlmoos T Yue D Chen J Benharash P Franzini L. Does universal insurance influence disparities in high-quality hospital use for inpatient pediatric congenital heart defect care within the first year of diagnosis? BMC Health Serv Res. (2023) 23(1):702. 10.1186/s12913-023-09668-1 37381049 PMC10303295

[19] Chan T Barrett CS Tjoeng YL Wilkes J Bratton SL Thiagarajan RR. Racial variations in extracorporeal membrane oxygenation use following congenital heart surgery. J Thorac Cardiovasc Surg. (2018) 156(1):306–15. 10.1016/j.jtcvs.2018.02.103 29681396

[20] Chan T Lion KC Mangione-Smith R. Racial disparities in failure-to-rescue among children undergoing congenital heart surgery. J Pediatr. (2015) 166(4):812–818.e4. 10.1016/j.jpeds.2014.11.020 25556012

[21] Pasquali SK He X Jacobs JP Jacobs ML O'Brien SM Gaynor JW. Evaluation of failure to rescue as a quality metric in pediatric heart surgery: an analysis of the STS congenital heart surgery database. Ann Thorac Surg. (2012) 94:573–80. 10.1016/j.athoracsur.2012.03.065 22633496 PMC3828205

[22] Peterson JK Catton KG Setty SP. Healthcare disparities in outcomes of a metropolitan congenital heart surgery center: the effect of clinical and socioeconomic factors. J Racial Ethn Health Disparities. (2018) 5(2):410–21. 10.1007/s40615-017-0384-7 28849382

[23] Vashist S Dudeck BS Sherfy B Rosenthal GL Chaves AH. Neighborhood socioeconomic status and length of stay after congenital heart disease surgery. Front Pediatr. (2023) 11:11. 10.3389/fped.2023.1167064 PMC10390779 37534195

[24] DeMone JA Gonzalez PC Gauvreau K Piercey GE Jenkins KJ. Risk of death for medicaid recipients undergoing congenital heart surgery. Pediatr Cardiol. (2003) 24(2):97–102. 10.1007/s00246-002-0243-z 12360394

[25] Erickson LC Wise PH Cook EF Beiser A Newburger JW. The impact of managed care insurance on use of lower-mortality hospitals by children undergoing cardiac surgery in California. Pediatrics. (2000) 105(6):1271–8. 10.1542/peds.105.6.1271 10835068

[26] Tweddell JS Sleeper LA Ohye RG Williams IA Mahony L Pizarro C. Intermediate-term mortality and cardiac transplantation in infants with single-ventricle lesions: risk factors and their interaction with shunt type. J Thorac Cardiovasc Surg. (2012) 144(1):152–159.e2. 10.1016/j.jtcvs.2012.01.016 22341427 PMC3359406

[27] Sengupta A Bucholz EM Gauvreau K Newburger JW Schroeder M Kaza AK. Impact of neighborhood socioeconomic status on outcomes following first-stage palliation of single ventricle heart disease. J Am Heart Assoc. (2023) 12(6):e026764. 10.1161/JAHA.122.026764 36892043 PMC10111557

[28] Tillman AR Colborn KL Scott KA Davidson AJ Khanna A Kao D. Associations between socioeconomic context and congenital heart disease related outcomes in adolescents and adults. Am J Cardiol. (2021) 139:105–15. 10.1016/j.amjcard.2020.10.040 33203514

[29] Kolwaite AR Edwards JA Higgins M Kandaswamy S Orenstein E Boughton D. Associations between child opportunity index and pediatric cardiac surgical outcomes. J Pediatr. (2024) 270:114000. 10.1016/j.jpeds.2024.114000 38432295

[30] Mayourian J Brown E Javalkar K Bucholz E Gauvreau K Beroukhim R. Insight into the role of the child opportunity index on surgical outcomes in congenital heart disease. J Pediatr. (2023) 259:113464. 10.1016/j.jpeds.2023.113464 37172810

[31] Duong SQ Elfituri MO Zaniletti I Ressler RW Noelke C Gelb BD. Neighborhood childhood opportunity, race/ethnicity, and surgical outcomes in children with congenital heart disease. J Am Coll Cardiol. (2023) 82(9):801–13. 10.1016/j.jacc.2023.05.069 37612012

[32] Bucholz EM Sleeper LA Goldberg CS Pasquali SK Anderson BR Gaynor JW. Socioeconomic status and long-term outcomes in single ventricle heart disease. Pediatrics. (2020) 146(4):e20201240. 10.1542/peds.2020-1240 32973120 PMC7546087

[33] Ghanayem NS Allen KR Tabbutt S Atz AM Clabby ML Cooper DS. Interstage mortality after the Norwood procedure: results of the multicenter single ventricle reconstruction trial. J Thorac Cardiovasc Surg. (2012) 144(4):896–906. 10.1016/j.jtcvs.2012.05.020 22795436 PMC3985484

[34] Taylor LC Burke B Donohue JE Yu S Hirsch-Romano JC Ohye RG. Risk factors for interstage mortality following the Norwood procedure: impact of sociodemographic factors. Pediatr Cardiol. (2016) 37(1):68–75. 10.1007/s00246-015-1241-2 26260093

[35] de Loizaga SR Schneider K Beck AF Zang H Ollberding N Tweddell JS. Socioeconomic impact on outcomes during the first year of life of patients with single ventricle heart disease: an analysis of the National Pediatric Cardiology Quality Improvement Collaborative Registry. Pediatr Cardiol. (2022) 43(3):605–15. 10.1007/s00246-021-02763-2 34718855

[36] Spigel ZA Kalustian AB Zink J Binsalamah ZM Caldarone CA. Low parental socioeconomic position results in longer post-Norwood length of stay. J Thorac Cardiovasc Surg. (2022) 163(5):1604–1611.e1. 10.1016/j.jtcvs.2021.09.075 34952706

[37] Schneider K De Loizaga S Beck AF Morales DLS Seo JD Divanovic A. Socioeconomic influences on outcomes following congenital heart disease surgery. Pediatr Cardiol. (2024) 45(5):1072–8. 10.1007/s00246-024-03451-7 38472658 PMC11056327

[38] Sengupta A Gauvreau K Bucholz EM Newburger JW Del Nido PJ Nathan M. Contemporary socioeconomic and childhood opportunity disparities in congenital heart surgery. Circulation. (2022) 146:1284–96. 10.1161/CIRCULATIONAHA.122.060030 36164982

[39] Olugbuyi O Smith C Kaul P Dover DC Mackie AS Islam S. Impact of socioeconomic status and residence distance on infant heart disease outcomes in Canada. J Am Heart Assoc. (2022) 11(18):e026627. 10.1161/JAHA.122.026627 36073651 PMC9683652

[40] Steurer MA McCulloch C Santana S Collins JW Branche T Costello JM. Disparities in 1-year-mortality in infants with cyanotic congenital heart disease: insights from contemporary national data. Circ Cardiovasc Qual Outcomes. (2023) 16(7):e009981. 10.1161/CIRCOUTCOMES.122.009981 37463254

[41] Morris SA Ethen MK Penny DJ Canfield MA Minard CG Fixler DE. Prenatal diagnosis, birth location, surgical center, and neonatal mortality in infants with hypoplastic left heart syndrome. Circulation. (2014) 129(3):285–92. 10.1161/CIRCULATIONAHA.113.003711 24135071 PMC7302400

[42] Udine ML Evans F Burns KM Pearson GD Kaltman JR. Geographical variation in infant mortality due to congenital heart disease in the USA. Lancet Child Adolesc Health. (2021) 5(7):483–90. 10.1016/S2352-4642(21)00105-X 34051889

[43] Mackie AS Gauvreau K Newburger JW Mayer JE Erickson LC. Risk factors for readmission after neonatal cardiac surgery. Ann Thorac Surg. (2004) 78(6):1972–8. 10.1016/j.athoracsur.2004.05.047 15561011

[44] Lushaj EB Hermsen J Leverson G MacLellan-Tobert SG Nelson K Amond K. Beyond 30 days: analysis of unplanned readmissions during the first year following congenital heart surgery. World J Pediatr Congenit Heart Surg. (2020) 11(2):177–82. 10.1177/2150135119895212 32093562

[45] Demianczyk AC Behere SP Thacker D Noeder M Delaplane EA Pizarro C. Social risk factors impact hospital readmission and outpatient appointment adherence for children with congenital heart disease. J Pediatr. (2019) 205:35–40.e1. 10.1016/j.jpeds.2018.09.038 30366772 PMC6527093

[46] Kim YY He W MacGillivray TE Benavidez OJ. Readmissions after adult congenital heart surgery: frequency and risk factors. Congenit Heart Dis. (2017) 12(2):159–65. 10.1111/chd.12433 27992675

[47] Lopez KN Baker-Smith C Flores G Gurvitz M Karamlou T Nunez Gallegos F. Addressing social determinants of health and mitigating health disparities across the lifespan in congenital heart disease. J Am Heart Assoc. (2022) 11:e025358. 10.1161/JAHA.122.025358 35389228 PMC9238447

[48] Krishnan A Jacobs MB Morris SA Peyvandi S Bhat AH Chelliah A. Impact of socioeconomic status, race and ethnicity, and geography on prenatal detection of hypoplastic left heart syndrome and transposition of the great arteries. Circulation. (2021) 143(21):2049–60. 10.1161/CIRCULATIONAHA.120.053062 33993718 PMC8162295

[49] Peiris V Singh TP Tworetzky W Chong EC Gauvreau K Brown DW. Association of socioeconomic position and medical insurance with fetal diagnosis of critical congenital heart disease. Circ Cardiovasc Qual Outcomes. (2009) 2(4):354–60. 10.1161/CIRCOUTCOMES.108.802868 20031861

[50] Henize AW Beck AF Klein MD Adams M Kahn RS. A road map to address the social determinants of health through community collaboration. Pediatrics. (2015) 136(4):e993–e1001. 10.1542/peds.2015-0549 26391941

[51] Schidlow DN Anderson JB Klitzner TS Beekman III RH Jenkins KJ Kugler JD. Variation in interstage outpatient care after the Norwood procedure: a report from the Joint Council on Congenital Heart Disease National Quality Improvement Collaborative. Congenit Heart Dis. (2011) 6(2):98–107. 10.1111/j.1747-0803.2011.00509.x 21426523

[52] Stagg A Giglia TM Gardner MM Offit BF Fuller KM Natarajan SS. Initial experience with telemedicine for interstage monitoring in infants with palliated congenital heart disease. Pediatr Cardiol. (2023) 44(1):196–203. 10.1007/s00246-022-02993-y 36050411 PMC9436461

[53] Shustak RJ Huang J Tam V Stagg A Giglia TM Ravishankar C. Neighborhood social vulnerability and interstage weight gain: evaluating the role of a home monitoring program. J Am Heart Assoc. (2023) 12(18):e030029. 10.1161/JAHA.123.030029 37702068 PMC10547291

[54] Cherestal B Erickson LA Noel-MacDonnell JR Shirali G Graue Hancock HS Aly D. Association between remote monitoring and interstage morbidity and death in patients with single-ventricle heart disease across socioeconomic groups. J Am Heart Assoc. (2023) 12(23):e031069. 10.1161/JAHA.123.031069 38014668 PMC10727312

[55] Moore EF Gephart SM. State of the science of care coordination, rurality, and well-being for infants with single ventricle heart disease in the interstage period, an integrative review. Heart Lung. (2021) 50(5):720–9. 10.1016/j.hrtlng.2021.04.014 34107397

[56] Hanke SP Joy B Riddle E Ravishankar C Peterson LE King E. Risk factors for unanticipated readmissions during the interstage. Semin Thorac Cardiovasc Surg. (2016) 28(4):803–14. 10.1053/j.semtcvs.2016.08.011 28417868

[57] Michielon G DiSalvo G Fraisse A Carvalho JS Krupickova S Slavik Z. In-hospital interstage improves interstage survival after the Norwood stage 1 operation. Eur J Cardiothorac Surg. (2020) 57(6):1113–21. 10.1093/ejcts/ezaa074 32236554

[58] Ahmed H Anderson JB Bates KE Fleishman CE Natarajan S Ghanayem NS. Development of a validated risk score for interstage death or transplant after stage I palliation for single-ventricle heart disease. J Thorac Cardiovasc Surg. (2020) 160(4):1021–30. 10.1016/j.jtcvs.2019.11.001 31924360

[59] Simeone RM Oster ME Cassell CH Armour BS Gray DT Honein MA. Pediatric inpatient hospital resource use for congenital heart defects. Birt Defects Res A Clin Mol Teratol. (2014) 100(12):934–43. 10.1002/bdra.23262 PMC4422978 24975483

[60] Cherestal B Hudson Z Lopez KN. Applying interventions to address the social determinants of health and reduce health disparities in congenital heart disease patients. Curr Cardiovasc Risk Rep. (2022) 16(12):241–8. 10.1007/s12170-022-00710-1

[61] Mackie AS Ionescu-Ittu R Pilote L Rahme E Marelli AJ. Hospital readmissions in children with congenital heart disease: a population-based study. Am Heart J. (2008) 155(3):577–84. 10.1016/j.ahj.2007.11.003 18294499

[62] Kim ME Kasparian NA Zang H Pater C Chlebowski MM Marcuccio E. Are parent discharge readiness scores effective for patients with congenital heart disease after cardiac surgery? J Pediatr. (2023) 257:113361. 10.1016/j.jpeds.2023.02.008 36841508

[63] Reddy KP Jones AL Naim MY Mercer-Rosa L. Association of race and ethnicity with resource utilization among children with CHD: an evaluation of the National Health Interview Survey, 2010–2018. Cardiol Young. (2023) 33(8):1471–3. 10.1017/S1047951123000033 36636921

[64] Gurvitz MZ Inkelas M Lee M Stout K Escarce J Chang R-K. Changes in hospitalization patterns among patients with congenital heart disease during the transition from adolescence to adulthood. J Am Coll Cardiol. (2007) 49(8):875–82. 10.1016/j.jacc.2006.09.051 17320746

[65] Griffey RT Kennedy SK McGownan L Goodman M Kaphingst KA. Is low health literacy associated with increased emergency department utilization and recidivism? Acad Emerg Med. (2014) 21(10):1109–15. 10.1111/acem.12476 25308133 PMC4626077

[66] Agarwal S Sud K Khera S Kolte D Fonarow GC Panza JA. Trends in the burden of adult congenital heart disease in US emergency departments. Clin Cardiol. (2016) 39(7):391–8. 10.1002/clc.22541 27079279 PMC6490840

[67] Harbison AL Grady S Chi K Fernandes SM. Provision of transition education and referral patterns from pediatric cardiology to adult cardiac care. Pediatr Cardiol. (2016) 37(2):232–8. 10.1007/s00246-015-1267-5 26385471

[68] Flores G Bridon C Torres S Perez R Walter T Brotanek J. Improving asthma outcomes in minority children: a randomized controlled trial of parent mentors. Pediatrics. (2009) 124:1522–32. 10.1542/peds.2009-0230 19948624

[69] Flores G Lin H Walker C Lee M Currie JM Allgeyer R. Parent mentors and insuring uninsured children: a randomized controlled trial. Pediatrics. (2016) 137:e20153519. 10.1542/peds.2015-3519 27244706 PMC4811315

[70] Centers for Medicare and Medicaid Services Insurekidsnow.gov. Engaging parent mentors to increase participation of eligible children in Medicaid and CHIP. Available online at: https://www.insurekidsnow.gov/webinars-videos/webinars/20181108/index.html

[71] Centers for Medicare and Medicaid Services Insurekidsnow.gov. 2019 HEALTHY KIDS outreach and enrollment cooperative agreements. CMS awards $48 million to help enroll eligible children in health coverage. Available online at: https://www.insurekidsnow.gov/campaign/funding/2019-healthykids/index.html

[72] Centers for Medicare and Medicaid Services Insurekidsnow.gov. Connecting Kids to Coverage Outreach and Enrollment Grants. CMS Awards Nearly $6 Million in Funding for Nine New Connecting Kids to Coverage HEALTHY KIDS American Indian and Alaskan Native (AI/AN) 2020 Outreach and Enrollment Cooperative Agreements. Available online at: https://www.insurekidsnow.gov/campaign-information/outreach-enrollment-grants/index.html

[73] US Congress. Rules Committee Print 115-55. Text of Extension of Continuing Appropriations Act, 2018[Showing the text of H.J. Res 125, as introduced. Available online at: http://docs.house.gov/billsthisweek/20180115/BILLS-115SAHR195-RCP115-55.pdf

[74] Joynt Maddox KE Reidhead M Hu J Kind AJH Zaslavsky AM Nagasako EM. Adjusting for social risk factors impacts performance and penalties in the hospital readmissions reduction program. Health Serv Res. (2019) 54(2):327–36. 10.1111/1475-6773.13133 30848491 PMC6407348

