# Supplementary material for: Impact of social determinants of health on perioperative and interstage outcomes in congenital heart surgery a systematic review
Source: Front Cardiovasc Med. 2026 Sep 29;13:1881839. doi: 10.3389/fcvm.2026.1881839 (PMC13658589; doi:10.3389/fcvm.2026.1881839)
Supplement: Supplementary file 1 [file Datasheet1.docx]

**SUPPLEMENTARY MATERIAL 1**

**Detailed Search Strategies**

**A Contemporary Systematic Review on the Impact of Social Determinants of Health on Perioperative and Interstage Outcomes Following Congenital Heart Surgery.**

Sara Sakowitz MD MPH MBA^a,b^, Jennifer S. Nelson MD MS^c,d^, Justin Robinson MD^e^, Camilla S. Rossi MD^f^, David M. Shahian MD^g^, David T. Cooke MD^h^, Jeffrey P. Jacobs MD^i^, Shelby Aughtman MD^j^, Megan Schultz MD^k^, Meena Nathan MD MPH^l,m^, Claude Beaty MD^n^, Michal Shafer MD^o^, Deborah Kozik DO^p^, Glenn J. Pelletier MD MBA^q^

**Affiliations:** aDepartment of Surgery, University of California, Los Angeles, CA; bDepartment of Surgery, Massachusetts General Hospital, Boston, MA; cDepartment of Cardiovascular Services, Nemours Children’s Hospital, Orlando, FL; dDepartment of Surgery, University of Central Florida College of Medicine, Orlando, FL; eDivision of Cardiothoracic Surgery, University of Maryland School of Medicine, Baltimore, MD; fDepartment of Cardiothoracic Surgery, Weill Cornell Medicine, New York, NY; gDivision of Cardiac Surgery, Department of Surgery, Massachusetts General Hospital, Harvard Medical School, Boston, MA; hDivision of General Thoracic Surgery, University of California, Davis, Sacramento, CA; iDivision of Cardiovascular Surgery, Department of Surgery, University of Florida, Gainesville, FL; jDepartment of Surgery, Macon & Joan Brock Virginia Health Sciences at Old Dominion University, Norfolk, VA; kDepartment of Cardiac Surgery, University of Michigan, Ann Arbor, MI; lDepartment of Cardiac Surgery, Boston Children’s Hospital, Boston, MA; mDepartment of Surgery, Harvard Medical School, Boston, MA; nNemours Children’s Health, Delaware Valley, Wilmington, DE; oDivision of Cardiothoracic Surgery, University of Utah, Salt Lake City, UT; pDepartment of Cardiovascular and Thoracic Surgery, University of Louisville, KY; qDepartment of Cardiothoracic Surgery, Stanford School of Medicine, Stanford, CA

**Corresponding Author:**

Glenn J. Pelletier MD MBA	[gjpell@stanford.edu](mailto:gjpell@stanford.edu)

**Contents**

[**Search Strategy: PubMed**	3](#_Toc201504144)

[**Search Strategy: SCOPUS**	4](#_Toc201504145)

[**Search Strategy: CINAHL**	5](#_Toc201504146)

[**Search Strategy: PSYCINFO**	6](#_Toc201504147)

[**Search Strategy: Cochrane**	7](#_Toc201504148)

# **Search Strategy: PubMed**

**(("Congenital heart"[tiab] OR "Heart Defects, Congenital"[Mesh]) AND ("surg*"[tiab] OR "surgery"[tiab] OR "surgical procedure"[tiab] OR "cardiac surg*"[tiab] OR "Cardiac Surgical Procedures"[Mesh] OR "heart transplantation*"[Mesh] OR "heart transplant*"[tiab] OR "cardiac transplant*"[tiab])) OR ("Heart Defects, Congenital/surgery"[Mesh] OR "Congenital heart"[tiab] OR "Heart Defects, Congenital"[Mesh]) OR ("Heart Transplantation"[MeSH Major Topic]) AND ("social determinants of health"[tiab] OR "SDOH"[tiab] OR "disparit*"[tiab] OR "equity"[tiab] OR "socioeconomic"[tiab] OR "Healthcare Disparities*"[Mesh] OR "Social Class"[Mesh] OR "Socioeconomic Factors"[Mesh])** ((address[Filter] OR autobiography[Filter] OR bibliography[Filter] OR biography[Filter] OR booksdocs[Filter] OR casereports[Filter] OR classicalarticle[Filter] OR clinicalconference[Filter] OR clinicalstudy[Filter] OR clinicaltrial[Filter] OR clinicaltrialphasei[Filter] OR clinicaltrialphaseii[Filter] OR clinicaltrialphaseiii[Filter] OR clinicaltrialphaseiv[Filter] OR comparativestudy[Filter] OR congress[Filter] OR consensusdevelopmentconference[Filter] OR consensusdevelopmentconferencenih[Filter] OR controlledclinicaltrial[Filter] OR correctedandrepublishedarticle[Filter] OR dataset[Filter] OR dictionary[Filter] OR directory[Filter] OR electronicsupplementarymaterials[Filter] OR englishabstract[Filter] OR evaluationstudy[Filter] OR festschrift[Filter] OR governmentpublication[Filter] OR guideline[Filter] OR historicalarticle[Filter] OR introductoryjournalarticle[Filter] OR lecture[Filter] OR legalcase[Filter] OR meta-analysis[Filter] OR multicenterstudy[Filter] OR observationalstudy[Filter] OR overall[Filter] OR patienteducationhandout[Filter] OR periodicalindex[Filter] OR personalnarrative[Filter] OR practiceguideline[Filter] OR pragmaticclinicaltrial[Filter] OR publishederratum[Filter] OR randomizedcontrolledtrial[Filter] OR researchsupportamericanrecoveryandreinvestmentact[Filter] OR researchsupportnihextramural[Filter] OR researchsupportnihintramural[Filter] OR researchsupportnonusgovt[Filter] OR researchsupportusgovtnonphs[Filter] OR researchsupportusgovtphs[Filter] OR researchsupportusgovernment[Filter] OR review[Filter] OR scientificintegrityreview[Filter] OR systematicreview[Filter] OR twinstudy[Filter] OR validationstudy[Filter] OR videoaudiomedia[Filter] OR webcast[Filter]) AND (1990:3000/12/12[pdat]) AND (english[Filter]))

# **Search Strategy: SCOPUS**

TITLE-ABS ( ( "Congenital heart" ) AND ( "surg*" OR "surgical procedure" OR "cardiac surg*" OR "heart transplant*" OR "cardiac transplant*" ) ) OR TITLE-ABS ( "Congenital heart" ) AND TITLE-ABS ( "social determinants of health" OR "SDOH" OR "disparit*" OR "equity" OR "socioeconomic" OR "Social Class" ) AND PUBYEAR > 1989 AND PUBYEAR < 2025 AND ( EXCLUDE ( DOCTYPE , "ed" ) OR EXCLUDE ( DOCTYPE , "le" ) OR EXCLUDE ( DOCTYPE , "ch" ) ) AND ( LIMIT-TO ( LANGUAGE , "English" ) )**–**

# **Search Strategy: CINAHL**

((TI "Congenital Heart" OR AB "Congenital Heart" OR MH “Heart Defects, Congenital+”) AND ((TI “surg*” OR AB “surg*”) OR (TI “cardiac surg*” OR AB “cardiac surg*”) OR (MH “Cardiac Surgery+”) OR (MH “Heart Transplantation”) OR (TI “heart transplant*” OR AB “heart transplant*”) OR (TI “cardiac transplant*” OR AB “cardiac transplant*”)) OR ((MH “Cardiac Surgery+”) OR (TI "Congenital Heart" OR AB "Congenital Heart") OR (MH “Heart Defects, Congenital”) OR (MM “Heart Transplantation”)) AND ((“social determinants of health” OR “SDOH” OR “disparit*” OR “equity” OR “socioeconomic” OR MM “Healthcare Disparities” OR MM “Social Class” OR MM “Socioeconomic Factors”))

# **Search Strategy: PSYCINFO**

(((("Congenital heart disease" or "Congenital Heart Defects") and ("surg*" or "surgical procedure" or "cardiac surg*" or "heart transplant*" or "cardiac transplant*")) or "congenital heart") and ("social determinants of health" or "SDOH" or "disparit*" or "equity" or "socioeconomic" or "Social Class" or "Socioeconomic Factors")).mp. [mp=title, abstract, heading word, table of contents, key concepts, original title, tests & measures, mesh word]

# **Search Strategy: Cochrane**

"social determinants of health" or "SDOH" or disparit? or "equity" or "socioeconomic" or "Social Class" or "Socioeconomic Factors" in Title Abstract Keyword AND (("Congenital heart disease" or "Congenital Heart Defects") and (surg? or "surgical procedure" or cardiac surg? or "heart transplant*" or cardiac transplant?)) or "congenital heart" in Title Abstract Keyword
